# New Methods of Treating Locomotor Ataxia

**Published:** 1889-05

**Authors:** 


### NEW METHODS OF TREATING LOCOMOTOR ATAXIA.

Dr. Stembo, in a late number of the *Berliner klinische Wochenschrift*, has an interesting paper on the causation and treatment of locomotor ataxia. He is of the opinion, now so rapidly gaining ground, that a large majority of these cases have a syphilitic history. Among thirty-nine cases treated by him, twenty-four [61.6 per cent.] have had a syphilitic history of from eight to twenty-four years standing. Many of these cases had no other syphilitic symptom. One of his cases shows the difficulty which may occur in discovering syphilis. The patient denied ever having had the disease, and showed no traces of syphilis, but Dr. Stembo accidentally discovered that ten years before the man had been for some time a hospital patient under syphilitic treatment.

Notwithstanding his belief in the influence of syphilis in causing locomotor ataxia, he has never found any benefit from antisyphilitic treatment, where other symptoms of syphilis did not exist.

Like most other physicians, he has gone the rounds of the materia medica in the treatment of locomotor ataxia with little benefit. ELECTRICITY alone has been of use. He has used this agent in the form of the constant current—the induced current—and frictional electricity. Each one of these methods had a good influence, but acted in a somewhat different way from the others.

Frictional electricity produced the quickest results, especially in regard to sensibility and coördination; the constant current produced more lasting results; the induced current was midway between the other two. In order to secure the best results he has of late been using a combination of these as follows:

I. Constant and induced current after one another.

The anode, about seventy square centimeters in area, is attached somewhat sidewise on the neck, by a band. The cathode, a little smaller, is then applied "*stationsweise*" along the opposite side of the spinal column. The electrodes are then changed to opposite sides and the current applied in the same way as before. Three "stations" are used on each side, and for each station half a minute is allowed. The strength of the current is from 1 to 1½ milliampères.

He then attaches the neck electrode to the sternum and treats the back with the anode, especially the painful points or the place of the girdle feeling.

The anode is then attached to the electrode on the sternum and the other pole to a brush of forty square centimeters, and an induction current is applied. The current should never cause more than a slight tingling sensation. The brush is applied to the back, on both sides of the spinous processes, from above downwards, and is allowed to rest ten to fifteen seconds in a place.

II. Constant and induced current together.

The large electrode is fastened to the neck as before and connected with the anode of a galvano-faradic current, while the cathode, connected with the brush, is passed along the back as before.

The strength of the constant current is 1–1½ milliampères for the faradic 90.mm. R. A. of a normal induction apparatus, and for the same length of time.

In using static electricity the patient is placed upon an insulated table which is connected with the positive pole. The negative pole is connected with a common ball electrode and applied along the spine and to the painful places. In the beginning, the length of the spark must be very small; if the patient is sensitive the positive pole may be led to the earth.

The writer gives the histories of three cases as samples. In all these cases the symptoms were arrested by the treatment.

He drops the caution that care and considerable special knowledge in the use of the apparatus are needed in applying this treatment successfully.

In this connection it may be well to notice that Dr. Joseph Schreiber is authority for the statement [*Berliner klin. Wochenschrift;* 1888; No. 52.] that Dr. Freund, Docent in Vienna, has tried the Mitchell treatment—rest in bed, nutritious diet and massage—together with electricity, in a case of mild tabes. In addition to the ordinary diet, 1 to 1½ litres of milk were used every day.

The method of using the electricity is not stated.

The patient improved for a time, but the trouble recurred with all its original force and steadily grew worse.

The Suspension Treatment in this disease, first described by Motchowkowsky, but brought prominently before the profession by Professor Charcot, last winter, has been tried by Dr. Morton, as reported in the *New York Medical Record.* He uses a Sayre suspension apparatus. When the patients are able to use their hands he uses the head straps alone, allowing the patients to pull upon the rope with their hands raised high above the head. If strong enough, they may make the suspension alone; otherwise they are assisted.

When they cannot use their hands the arm-straps are used, the weight being divided equally between the head and arms. A convenient way in this last class of patients is to arrange the apparatus while the patient is seated in a chair. When all is ready, an attendant raises him to his feet whilst another pulls upon the rope.

He begins with a suspension of from one to two minutes, every second day, increasing the time to five or seven minutes. One suspension every second day is enough. The patients are raised so that the balls of the feet just touch the floor. The toes should not leave the floor, at least in self suspension. In helpless patients, it is a good plan to raise the arms occasionally so as to bring more weight upon the head.

Dr. Morton has tried the treatment in six cases, using over two hundred suspensions. So far, every case has improved under the treatment. The relief is almost immediate. He has never had any unpleasant results except in one case, when a patient "had a severe cramp like a 'crick' in the back of the neck. It lasted about a minute and soon wore off. The back strap of the apparatus against the neck probably pressed in upon the muscles already in a state of abnormal irritability. It should bear squarely upon the occiput."

Dr. Morton is hopeful as to the value of this treatment and thinks that "we shall be obliged to admit that the sum total of improvement and cure, be it temporary or permanent, is far in excess of that attainable by any previous means, and as such must be regarded as the most signal advance yet made in the treatment of this hitherto intractable disease."

